# Virtual and Augmented Reality Games in Dementia Care: Systematic and Bibliographic Review

**DOI:** 10.3390/healthcare13162013

**Published:** 2025-08-15

**Authors:** Martin Eckert, Varsha Radhakrishnan, Thomas Ostermann, Jan Peter Ehlers, Gregor Hohenberg

**Affiliations:** 1 Stabsstelle für Digitalisierung und Wissensmanagement, Hochschule Hamm-Lippstadt, 59063 Hamm, Germany; varsha.radhakrishnan@stud.hshl.de (V.R.); gregor.hohenberg@hshl.de (G.H.); 2 Fakultät für Gesundheit, Universität Witten-Herdecke, 58455 Witten, Germany; thomas.ostermann@uni-wh.de (T.O.); jan.ehlers@uni-wh.de (J.P.E.)

**Keywords:** medicine, dementia, Alzheimer’s, virtual reality, augmented reality, systematic review, bibliographic review

## Abstract

**Background:** This review investigates the use of virtual and augmented reality games in dementia care. It provides an insight into the last 13 years of research, including the earliest publications on this topic, and takes a systematic and bibliographic approach. **Methods:** We sourced research publications from three different scientific databases (PubMed, Scopus, and APA PsycInfo) for this publication. We chose the PRISMA approach and categorized the studies according to the publisher. A set of 12 variables was defined across three categories (bibliographic, medical, and technical). **Results:** Of the 389 identified articles, 36 met the inclusion and exclusion criteria. After a phase of pilot studies mainly being conducted, the number of publications increased by four times but decreased again in 2023. Dominating were pilot and feasibility studies; 8 out of the 36 trials were RCTs. The median trial population was 24, and the protocols were performed for an average of 10 weeks, with two 40-min sessions a week. Simulator sickness was reported but not by the majority of participants. A total of 59% of the studies used fully immersive 3D-VR systems. We identified only three publications that provided high immersion quality. These findings indicate the positive effects of using virtual and augmented reality systems on participants’ cognitive function and mood. **Conclusions:** This publication focuses on the technical aspects of the applied technologies and immersion levels of the patients. Using augmented and virtual reality methods to improve the quality of life and physical interaction of dementia patients shows the potential to enhance cognitive functioning in this population, but further investigation and multicenter RCTs are needed. There are strong indications that this research branch has high potential to benefit both caretakers and patients.

## 1. Introduction

Dementia and Alzheimer’s disease are significant challenges in Europe and worldwide, particularly as many Western societies are experiencing an aging population [1]. The rising prevalence of dementia-related disorders highlights the importance of developing innovative methods for providing routine care and a deeper understanding of this disease.

Treatment for dementia focuses on providing cognitive and physical stimulation to help slow the decline in neurocognitive functions. This review examines the connection between dementia treatment and emerging virtual and augmented reality technologies. It summarizes the current state of the research and its potential to improve patient outcomes and enhance well-being, helping to guide advancements in research, clinical applications, and technical design.

### 1.1. Background

Roberts and Knopman state that dementia involves a decrease in cognitive abilities, difficulties in thinking, and a diminished memory function capacity [2]. The WHO dementia report, based on GBD2019 data, estimates that there are 55.2 million people worldwide living with dementia. This is broken down by region: the Western Pacific Region has the highest population of dementia patients with 20.1 million, followed by the European Region with 14.1 million and the Americas with 10.3 million [3]. Newer WHO publications are projecting that the total number will reach 78 million by 2030, resulting in an economic cost of USD 2.8 trillion [4]. Video games offer interactive electronic experiences that can affect behavior, cognition, and emotions. This can have effects like improved attention and enhanced problem-solving as noted by Rodrigo-Aynguas et al. [5] in an attention deficit hyperactivity disorder (ADHD) context, as well as better emotional health, discussed by Pallavicini et al. within the scope of stress reduction [6].

Eckert et. al. reviewed the research landscape at the intersection of video games and dementia in an earlier publication [7]. This review is built upon the publication and applies a similar methodology with a narrowed focus on projects featuring virtual reality (VR) and augmented reality (AR).

VR and AR technologies have been applied successfully in other healthcare domains. For example, exergaming has been used to improve physical function, cognition, and depression in nursing home residents—see Peng et al. [8]—and Reminiscence Training was reviewed by Lu et al. [9]. When games go beyond simple entertainment, they are classified as serious games, often utilized in healthcare or education. A video game transitioning from solely cognitive engagement to physical activity is an exergame—a fusion of exercise and gameplay—a concept initially introduced by Bogost in 2005 [10].

VR and AR technologies have become more accessible, evolving from devices with niche applications to consumer-oriented entertainment product, as emphasized by Munoz-Saavedra et al. in 2020 [11]. VR immerses users in a wholly computer-generated environment. Conversely, AR overlays digital information onto real-world scenes to enhance the user’s perception of reality.

AR and VR allow users to explore every imaginable landscape, town, mountain, or forest in virtual environments with minimal real-world risk. A reduced hardware size, controller functionality enhancements, and better frame rates create a lower likelihood of motion sickness. Furthermore, AR and VR technologies can improve the physical and cognitive activity levels among People with Dementia (PwD). This review distinguishes itself by focusing on technical aspects such as content delivery, control methods, and the levels of immersion in augmented and virtual spaces. It serves the research community by guiding future initiatives and informing researchers about relevant technologies.

### 1.2. Overview

In 2022, Flynn et al. [12] examined the role of key stakeholders in implementing AR and VR solutions for older adults with dementia. Adopting a qualitative approach, they documented the experiences and perspectives of those involved in patient care. The review included 14 studies that revealed three analytical themes: ‘entering virtuality’, ‘a virtual world’, and ‘returning to reality’. The findings suggested that VR can provide positive experiences for older individuals with Mild Cognitive Impairment (MCI) and can have a tangible impact on their lives. Additionally, they emphasized the importance of designing VR interventions with great sensitivity and involving the intended recipients in the design process to ensure effective technology use in dementia care.

A publication by Yen et al. (2021) examined the effectiveness of virtual reality exergames in enhancing cognition and alleviating depression in older adults [13]. They analyzed eighteen randomized clinical trials, which revealed moderate effects on participants’ overall cognitive function and memory but significant effects on the depression outcomes. Interestingly, commercial VR games demonstrated a more substantial positive impact on the depression outcomes than VR-aided exergaming. The review concluded that there is potential to positively influence cognition, memory, and depression outcomes in older adults [13].

In an extensive review of 325 prescreened publications, Gates et al. [14] found that compared to active control interventions, computerized cognitive training led to a slight but not sustainable improvement in cognitive function after a 12-week training period was completed and the interventions were stopped. The study indicated that the amount of evidence available and its quality were too low to draw definitive conclusions. There were signs of a slight improvement in episodic memory, but the effects on working memory and verbal fluency were minor to negligible. Furthermore, the research available at the time lacked data on follow-ups, quality of life indicators, psychological well-being, daily functioning, and adverse events. The primary issues identified included imprecision and a risk of bias. Higher-quality evidence is necessary to reach conclusions with greater certainty [14].

Studies report positive effects on patients’ experiences, cognitive function, and physical activity. However, the supporting evidence remains limited. More research is needed on the use of virtual and augmented reality for treating dementia and other neurocognitive conditions, focusing on patient-centered outcomes that consider quality of life, well-being, and daily functionality.

## 2. Method

**Figure 1:**
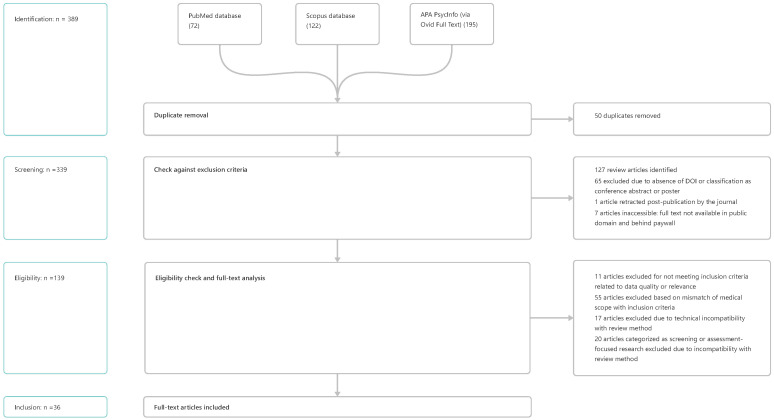
PRISMA workflow. A visual representation of the stages involved, illustrating the individual steps and the number of excluded publications at each stage.

The research design was developed using Moher et al.’s publication, with the Preferred Reporting Items for Systematic Reviews and Meta-Analyses (PRISMA) guidelines applied where applicable. The PRISMA Statement reports all the relevant items, and a visual overview is presented in Figure 1. The following section describes the research design.

### 2.1. Strategy for Article Search and Identification

This review sourced research from three major scientific databases: PubMed, Scopus, and APA PsycInfo. We refined the focus on treating dementia and Alzheimer’s disease to include exergames and video, computer, and serious games. This focus was explicitly fine-tuned to investigate gaming-based approaches and further limited to publications that applied virtual and augmented reality technology, as seen in the corresponding search query in Listing 1. Due to the design of the query mechanism, the query for the Scopus database had to be adapted but had the same scope; see Appendix A Listing A.1.

**Listing 1.** Search query for Pubmed and APA PsycInfo.
((dementia) OR (alzheimers disease)) AND

((video games) OR (computer games) OR (exergames) OR (serious games)) AND

((augmented reality) OR (virtual reality))


The team queried the three databases on 9 March 2025, retrieving an initial set of 389 results in total.

We converted the search results into a comma-separated file for each database to increase the readability and facilitate further investigation. Using the three files, the DOIs were extracted and imported into Zotero 7.0.16 library management software. Zotero 7.0.16 was used to obtain clean metadata. Furthermore, the software acted as a PDF and library management tool to access and annotate publications in the screening and extraction phase.

### 2.2. Article Screening and Eligibility

Following the initial screening of 389 publications, we identified 50 duplicates originating from an overlap between the three data sources. Review articles were excluded from this publication. A selection of the latest reviews is summarized in the Background Section 1.2. In total, 127 reviews were identified and excluded. After applying the exclusion criteria to the remaining result set, 139 articles were retained for manual screening and the application of the exclusion and eligibility criteria. The complete table of applied inclusion and exclusion criteria can be found in the appendix, Table A3. In total, 36 publications were included in the review after the screening and eligibility phases.

### 2.3. Scope of Investigation

Following the initial review and the application of the eligibility criteria, the 36 remaining publications underwent manual screening and a full-text analysis. Before starting this process, variables were defined and extracted from the publications and their available metadata. The following section describes the review design and variables.

#### 2.3.1. Bibliographic Variables

Bibliographic variables offered detailed insights into the publications. Two bibliographic variables proved to be significant for further investigation and visualization, see Table 1.

#### 2.3.2. Medical Variables

In a medical setting, factors such as the type of clinical trial, participant numbers, and engagement levels provide valuable insights into the research’s status and the effectiveness of the applied interventions, see Table 2.

#### 2.3.3. Technical Variables

The following variables were investigated in order to gain technical insight into the development and status of VR/AR applications. They are described in Table 3.

The **immersion level** variable was adapted from Milgram’s taxonomy of mixed reality [15], which involves a 3-axis continuum that creates a space for classifying VR and AR technologies.

A combination of the Extent of Presence (EP) and Reproduction Fidelity (RF) values was used to create a plane (x and y) to classify the overall immersion level. The results of this assessment were plotted on a grid as seen in the, Section 3.3.4. The authors’ definition of immersion level and its derivation from Milgram can be read in the Appendix A.3.

### 2.4. Data Presentation

We manually extracted the variables from the entire text for each publication. If a specific value was missing, we excluded only that value and its corresponding publication when considering the relevant variable while keeping it in the full dataset. We utilized a combination of violin and scatter plots to visualize complex datasets. All the figures were generated using Python 3.10 and the following libraries for data mining and visualization: Pandas 2.1.4, NumPy 1.24.3, Matplotlib 3.7.1, Seaborn 0.12.2, and Plotly 5.9.0.

## 3. Results

This section represents the results of the reviewed publications and gathered data. The full dataset can be acquired online at the OSF directory https://osf.io/cety3/ (accessed on 11 August 2025).

### 3.1. Bibliographic Results

This section presents the bibliographic results and features an analysis of the publication year and the corresponding author’s location.

#### 3.1.1. Timeline of Publications

**Figure 2:**
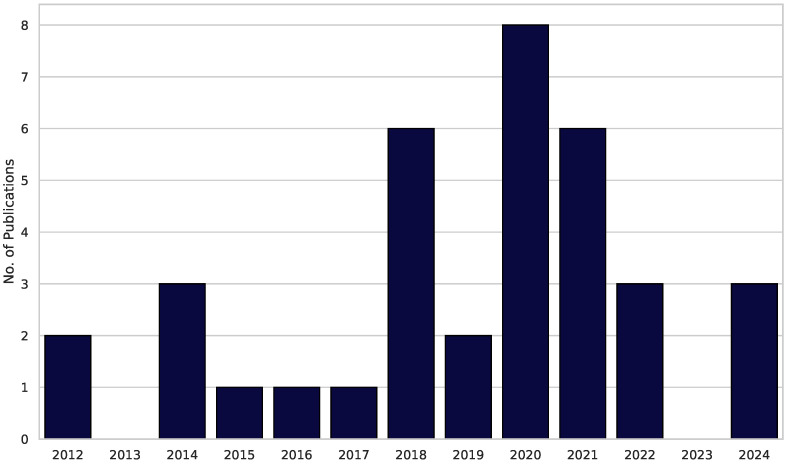
**Timeline of publications.** The bars illustrate the number of publications in each specific year, ranging from 2012 to 2024.

In total, we looked at 12 years of research and 36 publications. The first pioneers in this field were Anderson et al., who published a study in 2012 [16]. No earlier publications were identified while searching the three databases. The results are visualized in the timeline of publications in Figure 2.

The graph can be divided into three sections. There was an early phase of pioneering research from 2012 to 2017 with a focus on single cases, pilots, and simple 2D games. Between 2018 and 2021, the annual research output almost tripled. This can be explained by advancements in HMD hardware technology, like the commercial launch of HMDs (e.g., Oculus Rift and HTC Vive). First, the RCTs signal a methodological upgrade from the early phase. Post-COVID recruitment and potential funding realignments bottleneck the research in the third phase. After no scientific work was published in 2023, 2024 saw three more publications covering trials conducted in this research field.

#### 3.1.2. Country of Origin

The 36 publications’ countries of origin show that three major research hubs, dominated by Canada, Germany, and Greece, are the main clusters. These are followed by countries with a strong affinity for the med-tech industry, such as South Korea and Taiwan, which are also able to turn new hardware developments into clinical pilots. The remaining countries only appear once, likely due to funding barriers and the early-stage nature of the research field. For more information, refer to Table 4 showing the countries of origin.

### 3.2. Medical Results

This section focuses on the medical results and findings. The following information was considered: the level of advancement of clinical research, participation in clinical research, patient engagement intensity, and occurrence of simulator sickness.

#### 3.2.1. Level of Advancement of Clinical Research

The size of and participation in clinical research studies are suitable indicators of a research field’s maturity. The publications were classified as randomized clinical trials, pilot studies, feasibility studies, design studies, and single case studies, as explained in Section 2.3.2 on the medical variables.

Table 5 provides insight into the maturity of this research field and its projects. A third (26 out of 36 publications) was classified as pilot or feasibility studies, indicating that this research field is explorative and the interventions are being refined. The data points toward the finding that virtual/augmented-reality dementia research is still pre-clinical. Most research teams are still improving hardware, finding doses. Eight conducted randomized clinical trials are a signal of maturation, yet the small number hinders comprehensive clinical meta-analysis.

#### 3.2.2. Participation in Clinical Research

The participation numbers in clinical trials are essential in quantifying the significance, sample size, and reliability.

**Figure 3:**
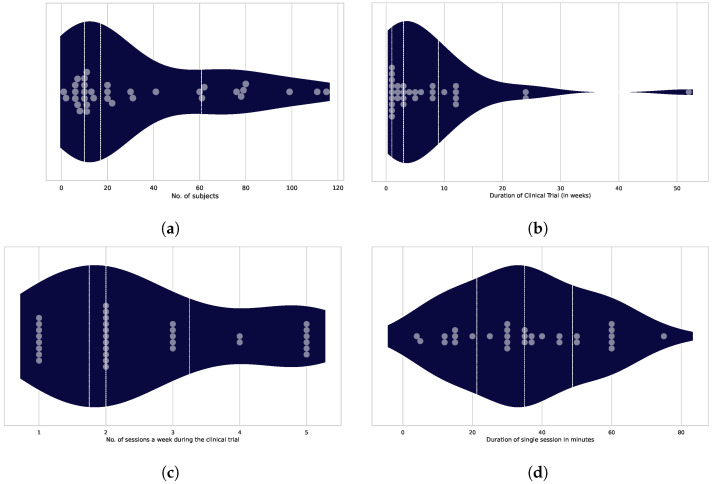
(**a**) **Number of subjects in the study:** The median is 16 subjects in 34 trials. The dots represent the individual trials and subject numbers. (**b**) **Overall duration of the study:** The average trial length was 3 weeks. The grey dots denote individual trials, while the dark blue violin plot shows the distribution of the number of publications over varying durations (n = 31). (**c**) **Number of sessions per week:** The average number of sessions per week was two sessions. The grey dots represent individual trials and the corresponding session counts. (**d**) **Single-session duration:** The median single-session duration is 35 min.

The participation numbers were visualized using a violin plot Figure 3a, supporting the results described in Section 3.2.1. With a median trial population of 34 subjects, it is evident that the use of VR is not yet part of any clinical routine. Even though there are outliers constituting well-populated trials like those conducted by Karssmeijer et al. [17] (115) and Anderson-Hanley et al. [18] (111), they remain sparse.

#### 3.2.3. Engagement Intensity

This section describes the intensity of engagement that the trial participants experienced. Three elements determine the engagement intensity of a subject during a session:

1. The overall duration of the subject’s participation in the trial, measured in weeks, as shown in Figure 3b.
2. The number of sessions per week conducted during the trial is shown in Figure 3c and referred to as the “dose”.
3. The typical length of one session, expressed in minutes, is shown in Figure 3d as the single-session duration.

The graphs below show variations in the overall publication numbers in relation to the following three variables. The descriptions of the three figures specify the total number of publications included in the analysis for each variable.

#### 3.2.4. Simulator Sickness

Of the thirty-six publications, six reported varying levels of simulator sickness during the trial, sixteen reported no occurrences of simulator sickness, and the remaining fourteen provided no information on the topic.

Zhu et al., who used an HMD to deliver a Virtual Supermarket Application, reported that one patient experienced simulator sickness, but this was resolved after the fifth session [19]. Amaefule et al., the team who developed the GRAIL System, a 180° walking simulator, in 2022 [20], identified that one out of thirteen subjects had problems with simulator sickness. Although this was a rare occurrence, these trials were classified as involving cases of simulator sickness.

### 3.3. Technical Results

This section presents the relevant technical data, covering the level of immersion, the hardware used for delivery (Extent of Presence: Delivery) and enabling interaction (Extent of Presence: Controls), and the production quality of the content (Reproduction Fidelity). Furthermore, we examine the types of immersion and determine whether a solution could be used autonomously by a patient or if guidance is required.

#### 3.3.1. Extent of Presence: Delivery

**Figure 4:**
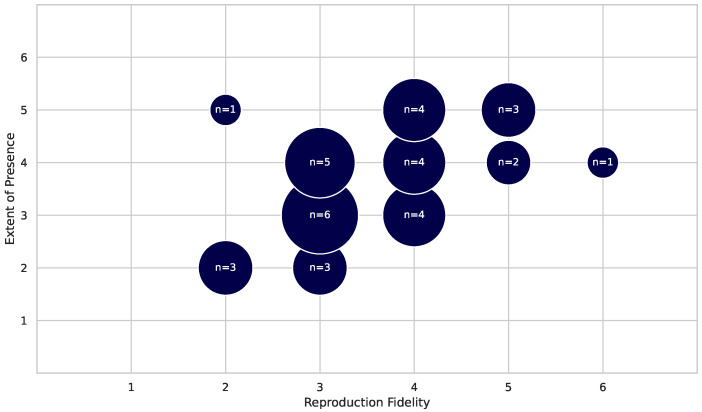
**Immersion level:** The Reproduction Fidelity (RF) is shown on the x-axis as an indicator of the graphical and auditive modeling quality. The Extent of Presence on the y-axis represents the users’ engagement with and immersion in the VR and AR worlds (n = 36).

How the virtual world is accessed by the patient is partly defined by the Computer Output Device. Milgram et al. describe the delivery method as the Extent of Presence (EP)  [15]. Regular TV screens and computer monitors have lower scores, and more advanced VR and AR systems have higher scores on the EP axis. The EP is shown along the y-axis in Figure 4. Table 6 shows the count of publications for each display type.

Information on the method of delivery was provided by all 36 publications (n = 36). We see a strong dominance of HMDs, parallel to the release of consumer-grade hardware starting in 2016. The application of HMD devices does have a positive effect on the EP sector of the immersion level; see also Figure 4. This indicates a more immersive experience for the user.

The German researchers Amaefule et al. built a walking simulator featuring a 180° field of view provided by a projector in front of an extra-wide treadmill [20]. The system’s benefits include its ease of use for the patient and great immersion without relying on HMDs to generate it. The surrounding 3D environment can be controlled using the walking speed.

#### 3.3.2. Extent of Presence: Controls

This variable, which influences the patient’s Extent of Presence in the virtual world, focuses on the hardware used to interact with the virtual environment. A versatile controller can significantly improve the user experience and create a more profound sense of immersion, while a simplistic controller can decrease immersion and accessibility. This variable was analyzed for all the publications (n = 36).

Table 7 shows the controllers used in the trials and their distribution.

The number of hand controllers was in alignment with the number of HMDs used, as the combination of an HMD and hand controller hardware is part of modern consumer-grade VR setups. The method of controlling a virtual avatar using hand controllers can be learned quickly and has a low setup time and cost.

Physical controllers, such as stationary bikes and treadmills, were used in six studies. They increase the sense of presence more than hand or game controllers, but their adoption seems to be hindered by cost factors, available spaces in care facilities, and supervision requirements.

Camera tracking devices have been piloted but often depend on the lighting conditions and are not as reliable as other input methods, so they remain sparsely used until they provide a more stable input method.

#### 3.3.3. Reproduction Fidelity

The Reproduction Fidelity (RF), as proposed in Milgram’s taxonomy [15], distinguishes between levels of quality regarding how virtual entities are modeled and rendered within VR and AR environments. Minimalist graphic and sound environments score low (1), and the most advanced real-time, realistic audio and video production scores high (6). All the results are displayed in Table 8.

The distribution is middle-loaded, as most applications featured mediocre quality in terms of their Reproduction Fidelity. Only 6 out of 36 publications delivered a state-of-the-art audiovisual experience. Four of the thirty-six were ranked below average. This variable was analyzed for all the publications (n = 36). Higher RF values, in theory, improve immersion and emotional engagement scores. Yet the small trial numbers prevent such analysis. The current mediocre plateau mirrors the field’s early research stages. Clinical investigators seem to prioritize the feasibility over immersive high-value productions.

#### 3.3.4. Immersion Level

The immersion level is essential to an application’s acceptance and effectiveness. A combination of the Extent of Presence (EP) and Reproduction Fidelity (RF) determined the publications’ locations in the grid, as these variables influence the immersion level of an AR/VR application. They are key factors in deciding how convincing the technology is for users and reflect how much a person feels like they are part of the virtual world.

In Figure 4, you can see how the immersion level is mapped out, with the RF on the x-axis and the EP on the y-axis. The scores ranges from 1 to 6. The visualization shows where different studies fall on the scale of immersion, with those near the upper right corner demonstrating high levels of both presence and fidelity.

Three clusters can be identified in the immersion level plot above. The core cluster (RF of 3–4, EP of 3–4) represents mediocre graphics and a mediocre presence in the virtual world. This indicates the widespread use of cost-saving hardware (e.g., HD TVs and common game controllers), which does not provide a deep feeling of immersion in the world. Out of 36 publications, 19 belong to this cluster. The second cluster (RF = 5, EP = 5+) represents the best in class. The technologies described in these publications provide excellent VR experiences and are most likely rare because of their high costs. Six publications belong to this cluster. The third cluster (RF <= 2, EP <= 2) is found at the lower end of the ratings. Only three publications provide low immersion and Reproduction Fidelity. This can be explained by the advancements in technology and our retrospective examination of older publications.

The research field is dominated by a mid-level-immersion dominance. Solutions need to be affordable and scalable but miss the mark in reaching maximal cognitive stimulation through high immersion. Only a handful of research groups penetrate the high immersion level, leaving a gap in evidence for future research.

#### 3.3.5. Immersion Type

In this category, 3D VR is the most common. Where the full distribution can be seen in Table 9 Immersive 3D HMDs are gaining traction and are the new methodical standard for research and pilot studies featuring virtual reality applications. A third of the publications used conventional methods (Monitor/tablet) with a lower risk of motion sickness and increased usability within an older population. Simple flat screens not showing virtual worlds have only been used in earlier proofs of concept, whereas the use of augmented reality has been limited to a single study, most likely because of the associated costs, unavailability of software, and usability issues.

The lack of research on AR/MR means there is a lack of data about interventions that manage to blend real-world experiences with augmented content.

#### 3.3.6. Autonomy and Guidance

This section covers whether supervision is necessary during the intervention. Ideally, a patient could use an application independently, resulting in more efficient care and lower treatment costs. Table 10 indicating a clear distribution of supervised projects. The benefit of using supervised solutions is that immediate assistance can be given to the patient, guiding them to perform the exercise in a consistent and ideal manner. Progress can be seen, and feedback on interventions and applications can be provided in real time. Furthermore, the patient might benefit from social interaction with the supervisor.

The majority of the reviewed publications used a supervised approach. Only one publication allowed the patients to use the application without supervision: van der Kolk et al. demonstrated the use of a cycling simulator called *Cybercycle*, which can be ridden autonomously [21].

## 4. Discussion

This systematic review explored the use of VR and AR technologies in new interventions for treating dementia.

### 4.1. Principal Findings

The publication rate increased 3-fold between 2018 and 2021, which can be explained by the breakthroughs in affordable VR hardware (the release of the Oculus CV1 in 2016, HTC Vive in 2016, Oculus Rift in 2019, and Quest 2 in 2019). It slowed during the COVID-era trough, due to recruitment and funding constraints. Research clusters can be identified in Canada, Germany, and Greece; however, the sample size is too small to draw further conclusions.

A total of 72% of the publications were classified as pilot or feasibility studies. Eight studies were classified as RCTs, but no RCT was identified that utilized a fully immersive 3D VR setup. Only one study, which had a population of 99, used an HMD [22]. While high-quality non-randomized trials reflect the nature of the iterative-technical approach seen in many trials with technology aids, randomization is valuable but not a sole indication for maturity. The use of applications employing high-EP and high-RF VR in clinical practice is still in an exploratory phase. At the same time, the setups changed with the ever-evolving hardware, and we identified a lack of clinical applications compared to research pilots. This finding was supported by a median of 34 subjects being included, on average (n = 34), which is statistically insufficient evidence to detect effects in the study populations.

From 2012 to 2017, no HMDs were used due to their unavailability on the consumer market. The first studies using the Oculus Rift CV 1 were published in 2017. Since then, this type of hardware has improved in terms of its field of view, resolution, usability, and weight, probably leading to an improvement in simulator sickness occurrences. Often mentioned as a hindrance to the use of 3D VR in clinical setups, only six out of thirty-six publications reported the occurrence of simulator sickness. These reports are not of clinical relevance, as they show that simulator sickness is occurring but not on a large scale. It is debatable whether there were no occurrences in the other trials or if they simply failed to report them.

Even though the search query included augmented reality and mixed reality applications, only a single study involving these could be identified [23]. The number of randomized clinical trials was lower than that of pilot and feasibility studies. This suggests that this area of research is slowly maturing and these technologies are finding clinical applications. The number of participants in the clinical trials, including those in the control groups, averaged 16 subjects.

There were almost no reports of simulator sickness, but it was under-reported. Only five of the nineteen publications contained information on simulator sickness, and none reported severe problems with it. Due to the complexity of VR and AR settings and state-of-the-art technology, their use is often not effortless. There was a clear tendency towards supervised interventions. While this field is still young, autonomous interventions will become easier to handle with advanced technology, and we might see a trend towards unsupervised applications.

The results demonstrated that patients’ level of immersion in the applications was average. Utilizing the MR continuum developed by Milgram et al. [15], we found that only a handful of applications were in the upper right sector and created a convincing level of immersion for the patient. Addressing this could directly improve patients’ quality of life and also help to enhance testing scores. Except for in one study, the use of all the applications had to be guided by a researcher or clinical personnel.

### 4.2. Strengths and Limitations

We limited the search to three Western databases; see Section 2.1. Studies from other countries (e.g., studies published in the CNKI database) may not have been considered in this publication due to accessibility issues and language barriers. Information was extracted through a manual screening process and application analysis, in contrast to using text mining approaches and solely analyzing metadata. During this process, there was an improbable chance of individual bias on the part of the screening authors. This was mitigated by having multiple authors act as screeners who extracted the same information and correlated the outcomes.

The focus of this review was of a technological nature, with a limited scope regarding the medical results and clinical outcomes. Future research should incorporate clinical evidence and, more importantly, examine the sustainability of the reported clinical results.

When planning larger RCTs, a six- to twelve-week follow-up period should be considered to assess long-term outcomes. Gates et al. reviewed the existing clinical evidence and practical implications with regard to planning RCTs on computerized cognitive training [14].

We understand that a combination of randomized trials and equally strong mixed-method approaches is needed to facilitate transparent reporting of clinical outcomes and potential biases. This would enable us to analyze future studies in terms of correlations between technological advancements in AR and VR devices, such as an increased field of view, resolution and frame rate, with the reported clinical outcomes. To achieve this, we recommend standardizing the reporting of hardware and software specifications to ensure that technological and clinical evidence are reported in the same way. Further, in terms of analyzed data quality, this review would benefit from a quality assessment for the individual studies and projects, which has not been considered in the initial method design.

The authors sought to establish correlations between the defined variables of the screened data, but not all the publications provided sufficient evidence to do so. Some publications fell short in their clinical outcome reports due to their nature as pilot studies or did not describe the applied technology in detail, often because they were conducted and written by healthcare professionals with a lesser focus on the technical aspects.

The immersion level classification method can be extended based on the FIVE Framework proposed by Slater and Wilbur [24] to examine further the technological aspects of the screened AR and VR technologies. This framework is based on the overall virtual presence of the user and possesses high potential for assessing the interface between psychology and the usage of AR and VR.

## 5. Conclusions

This text offers a perspective on the last 13 years of research on treating people with cognitive impairment using VR and AR games, which has been found to be beneficial and improve patients’ quality of life. Nonetheless, the evidence remains uncertain, highlighting the need to improve the technical aspects of these applications and conduct additional trials to understand their feasibility and functionality better. Further, the research landscape needs a standardized reporting method for technology specifications when used in a healthcare setting. We suggest incorporating six- or twelve-month follow-ups into RCTs to ensure a sustainable change in patients’ QoL and neurocognitive activation levels. To help plan this kind of research, we recommend conducting a multicenter randomized controlled trial (RCT) with more than 120 subjects and 6 months of weekly follow-up sessions to validate the long-term effects of immersive 3D-VR training. Also the collaboration with professional graphic and game studies or the adoption of generative-AI content pipelines may help to close the identified gap in fidelity. Clusters of research excellence in Germany and Greece, and the European Horizon 2025 call for proposals, offer potential funding options. With the ongoing advancements in hardware and software, VR and AR technologies can be integrated effectively into current workflows, enhancing treatments and offering new opportunities for patients, doctors, and caregivers to improve the quality of life of everyone involved.

## Figures and Tables

**Table 1:** Description of bibliographic variables.

Variable	Description
**Year of Publication**	The year the paper was first published.
**Country of Origin**	The publication’s country of origin was recorded to establish the geographic context. In cases where multiple authors and institutions were listed, the country corresponding to the first author’s primary affiliation was used.

**Table 2:** Clinical research variables and their descriptions.

Variable	Description
**Level of Advancement of Clinical Research**	The type of trial conducted and referenced in the evaluated publication.
Design Study	Small, controlled or sometimes uncontrolled intervention. It does not need to be randomized.
Pilot Study	A limited study is carried out to examine factors like the protocol’s usability, required duration, cost, and risks. It does not evaluate the treatment’s effects.
Feasibility Study	Evaluation of the feasibility of a clinical trial, featuring both randomization and control groups. This type of trial includes fewer participants than a randomized controlled trial (RCT), yet more than a pilot study, and is conducted in preparation for a larger RCT.
Randomized Clinical Trial (RCT)	Participants are randomly placed into intervention groups to assess the treatment’s impacts and risks compared to a control group.
**Participation in Clinical Research**	indicates the number of participants involved in a clinical trial or study.
**Engagement Intensity**	A collection of measures indicating the engagement intensity for the implemented interventions.
Overall Period of Engagement	Measured in weeks.
Number of Weekly Sessions	The total number of weekly sessions provided.
Average Session Duration	Duration of one session, expressed in minutes.
**Simulator Sickness**	Addresses the problem of simulator sickness in AR/VR applications, which arises from the disconnect between actual body movements and visual cues.

**Table 3:** Technical variables and their descriptions.

Variable	Description
**Extent of Presence: Delivery**	Refers to the type of device used to deliver content to the user.
AR	Digital content overlaid on the real world in real time using 2D devices.
HMD	Wearable display featuring stereoscopic 3D with motion tracking and spatial audio.
Laptop Screen	Standard 2D display. Limited field of view and spatial engagement.
TV/Monitor	Larger external 2D display, larger field of view than laptop.
Projector	Projects content onto a wall or flat surface for a larger field of view.
Tablet/Touchscreen	Interactive 2D device with touch input for interaction but limited immersion due to lack of spatial depth and limited field of view.
**Extent of Presence: Controls**	Refers to the type of controller used to interact with the application.
Game	Consumer-grade game controller, joystick.
Hand	Wireless handtracker, IR handtracker, VR handheld remote, touchscreen.
Physical	Treadmill, stationary bike.
Camera	Body movement tracker (e.g., Kinect).
Misc	Driving simulator, 3D-printed camera controller.
**RF: Reproduction Fidelity **	Rates the maturity and quality of the application on a scale from 1 to 6. A score of 1 represents simple sounds and graphics, while 6 represents a realistic real-time simulation.
**Immersion Level**	The Extent of Presence (EP) and Reproduction Fidelity (RP) values are used to classify the immersion level, plotted on a grid as seen in the Immersion Level subsection.
**Immersion Type**	Category of user engagement in the digital environment.
2D	This is 2D content rendered on a 2D screen (e.g., completing a quiz on a flat screen).
2D VR	Virtual reality content rendered on a 2D screen, tablet, or phone (e.g., biking in a virtual world on a 2D screen).
3D VR	Virtual reality content rendered inside VR headsets, with positional tracking, stereoscopic view, and interactive input.
AR/MR	Augmented reality and mixed reality: real-world surroundings with a virtual overlay.
**Autonomy and Guidance**	Represents the patient’s autonomy while using the application.
Supervised	The patient uses the application under supervision.
Unsupervised	The patient uses the application without supervision.

**Table 4:** Publications by country.

Country	Publications	Country	Publications	Country	Publications
Canada	8	Germany	5	Greece	4
USA	4	South Korea	2	Taiwan	2
Australia	1	Brazil	1	China	1
France	1	Italy	1	Malaysia	1
The Netherlands	1	Poland	1	Thailand	1
Turkey	1				

**Table 5:** Number of publications by study type.

Study Type	Publications
Design Study	2
Pilot Study	13
Feasibility Study	13
Randomized Clinical Trial (RCT)	8

**Table 6:** Number of publications by display type.

Display Type	Publications
HMD	17
TV	7
Projector	5
Computer Monitor	3
Laptop Screen	2
Tablet/Touchscreen	2

**Table 7:** Number of publications by controller type.

Controller Type	Publications
Hand	16
Game	7
Physical	6
Misc	4
Camera	3

**Table 8:** Aggregated Reproduction Fidelity (RF) scores.

RF	Number of Publications
1	0
2	4
3	14
4	12
5	5
6	1

**Table 9:** Number of publications by immersion type.

Immersion Type	Publications
2D	2
2D VR	11
3D VR	22
AR/MR	1

**Table 10:** Number of publications by supervision status.

Supervision Status	Publications
Supervised	35
Not Supervised	1

## Data Availability

This review and its protocol are registered in the OSF (Open Science Framework) with the ID gr2f3 (https://osf.io/gr2f3 (accessed on 11 August 2025)). The data is available from the OSF in a CSV format (https://osf.io/cety3 (accessed on 11 August 2025)).

## Abbreviations

The following abbreviations are used in this manuscript.

ADHD	Attention Deficit Hyperactivity Disorder
AR	Augmented reality
APA	American Psychological Association (preceding PsycInfo)
CNKI	China National Knowledge Infrastructure
EP	Extent of Presence
EWK	Extent of World Knowledge
GBD 2019	Global Burden of Disease 2019 study
HMD	Head-Mounted Display
ICD	International Classification of Diseases
MCI	Mild Cognitive Impairment
MeSH	Medical Subject Heading
MR	Mixed Reality
OSF	Open Science Framework
PRISMA	Preferred Reporting Items for Systematic Reviews and Meta-Analyses
PwD	People with Dementia
RCT	Randomized clinical trial
RF	Reproduction Fidelity
VR	Virtual reality
WHO	World Health Organization

## Appendix A. Table of Included Publications

**Table A1:** Table of the included trials, containing the number of subjects, year of publication, and device type. The publications are referenced accordingly.

Publication Title	Subjects	Device	Year
Exergaming as a Physical Exercise Strategy Reduces Frailty in People With Dementia: A Randomized Controlled Trial [17]	115	Projection Screen	2019
The Aerobic and Cognitive Exercise Study (ACES) for Community-Dwelling Older Adults With or At-Risk for Mild Cognitive Impairment (MCI): Neuropsychological, Neurobiological and Neuroimaging Outcomes of a Randomized Clinical Trial [18]	111	Monitor	2018
Effects of virtual reality-based cognitive training in older adults living without and with mild dementia: A pretest-posttest design pilot study [22]	99	HMD	2019
Wayfinding in aging and Alzheimer’s disease within a virtual senior residence: study protocol [25]	80	Projection Screen	2016
Exergaming and older adult cognition: A cluster randomized clinical trial [16]	79	Monitor	2012
Does cognition-specific computer training have better clinical outcomes than non-specific computer training? A single-blind, randomized controlled trial [26]	78	TV	2018
A Comparison of Traditional and Serious Game-Based Digital Markers of Cognition in Older Adults with Mild Cognitive Impairment and Healthy Controls [27]	76	Tablet/Touchscreen	2021
Everyday-like memory for objects in aging and Alzheimer’s disease assessed in a visually complex environment: The role of executive functioning and episodic memory [28]	62	Projection Screen	2016
The Effectiveness of a Virtual Reality-Based Intervention on Cognitive Functions in Older Adults with Mild Cognitive Impairment: A Single-Blind, Randomized Controlled Trial [29]	61	TV	2021
The Effectiveness of a Virtual Reality-Based Tai Chi Exercise on Cognitive and Physical Function in Older Adults with Cognitive Impairment [30]	60	TV	2018
Comparing data from a computer based intervention program for patients with Alzheimer’s disease [31]	41	Tablet/Touchscreen	2014
Immersive Virtual Reality-Based Cognitive Intervention for the Improvement of Cognitive Function, Depression, and Perceived Stress in Older Adults With Mild Cognitive Impairment and Mild Dementia: Pilot Pre-Post Study [19]	31	HMD	2022
Evaluation of a cognition-sensitive spatial virtual reality game for Alzheimer’s disease [32]	30	Laptop	2024
Enhancing prompt perception in dementia: a comparative study of mixed reality cue modalities [23]	22	HMD	2024
Treatment of Alzheimer’s, Cognitive, Chronic Pain Rehabilitation, Depression and Anxiety disorders in One System for Elderly Using VR [33]	20	Monitor	2018
A Virtual Reality App for Physical and Cognitive Training of Older People With Mild Cognitive Impairment: Mixed Methods Feasibility Study [34]	20	HMD	2021
The Long-term Effects of Immersive Virtual Reality Reminiscence in People With Dementia: Longitudinal Observational Study [35]	20	HMD	2022
2D Virtual Reality-Based Exercise Improves Spatial Navigation in Institutionalized Non-robust Older Persons: A Preliminary Data Report of a Single-Blind, Randomized, and Controlled Study [36]	14	TV	2021
Effect of Spatial Disorientation in a Virtual Environment on Gait and Vital Features in Patients with Dementia: Pilot Single-Blind Randomized Control Trial [20]	13	Projection Screen	2020
Lessons Learned from a Human-Centered Design of an Immersive Exergame for People with Dementia [37]	11	HMD	2021
Does Practicing with a Virtual Reality Driving Simulator Improve Spatial Cognition in Older Adults? A Pilot Study [38]	11	HMD	2020
Cognitive Training Using Fully Immersive, Enriched Environment Virtual Reality for Patients With Mild Cognitive Impairment and Mild Dementia: Feasibility and Usability Study [39]	11	HMD	2020
Feasibility study of the BrightBrainer™ integrative cognitive		Laptop	
rehabilitation system for elderly with dementia [40]	10	TV	2015
Virtual Reality for Persons with Dementia: an Exergaming Experience [41]	10	Projection Screen	2012
Effects of a virtual group cycling experience on people living with dementia: A mixed method pilot study [42]	10	TV	2020
A pilot study and brief overview of rehabilitation via virtual environment in patients suffering from dementia [43]	10	HMD	2018
Enabling Patients with Neurological Diseases to perform Motor-Cognitive Exergames under Clinical Supervision for Everyday Usage [44]	8	HMD	2020
Immersive Virtual Reality Exergames for Persons Living With Dementia: User-Centered Design Study as a Multistakeholder Team During the COVID-19 Pandemic [45]	7	HMD	2022
Using exergames to train patients with dementia to accomplish daily routines [46]	7	HMD	2020
Game Design for Users with Constraint: Exergame for Older Adults with Cognitive Impairment [47]	6	HMD	2018
Virtual Reality Exergames for People Living with Dementia Based on Exercise Therapy Best Practices [48]	6	HMD	2018
Participatory design and evaluation of virtual reality games to promote engagement in physical activity for people living with dementia [49]	6	HMD	2020
Development of Serious Game Theory Framework in Virtual Reality for Alzheimer’s Patients [50]	2	TV	2024
Two-week virtual reality training for dementia: Single case feasibility study [51]	1	HMD	2014
An Online Cognitive Intervention Tool for the Patients with Mild Cognitive Impairment using Virtual Reality [52]	not reported	HMD	2021
Memory Journalist: Creating Virtual Reality Exergames for the Treatment of Older Adults with Dementia [53]	not reported	not reported	2020

**Table A2:** Table of the included trials, containing the number of subjects, year of publication, and device type. The publications are referenced accordingly.

Publication Title	Subjects	Device	Year
Exergaming as a Physical Exercise Strategy Reduces Frailty in People With Dementia: A Randomized Controlled Trial [17]	115	Projection Screen	2019
The Aerobic and Cognitive Exercise Study (ACES) for Community-Dwelling Older Adults With or At-Risk for Mild Cognitive Impairment (MCI): Neuropsychological, Neurobiological and Neuroimaging Outcomes of a Randomized Clinical Trial [18]	111	Monitor	2018
Effects of virtual reality-based cognitive training in older adults living without and with mild dementia: A pretest-posttest design pilot study [22]	99	HMD	2019
Wayfinding in aging and Alzheimer’s disease within a virtual senior residence: study protocol [25]	80	Projection Screen	2016
Exergaming and older adult cognition: A cluster randomized clinical trial [16]	79	Monitor	2012
Does cognition-specific computer training have better clinical outcomes than non-specific computer training? A single-blind, randomized controlled trial [26]	78	TV	2018
A Comparison of Traditional and Serious Game-Based Digital Markers of Cognition in Older Adults with Mild Cognitive Impairment and Healthy Controls [27]	76	Tablet/Touchscreen	2021
Everyday-like memory for objects in aging and Alzheimer’s disease assessed in a visually complex environment: The role of executive functioning and episodic memory [28]	62	Projection Screen	2016
The Effectiveness of a Virtual Reality-Based Intervention on Cognitive Functions in Older Adults with Mild Cognitive Impairment: A Single-Blind, Randomized Controlled Trial [29]	61	TV	2021
The Effectiveness of a Virtual Reality-Based Tai Chi Exercise on Cognitive and Physical Function in Older Adults with Cognitive Impairment [30]	60	TV	2018
Comparing data from a computer based intervention program for patients with Alzheimer’s disease [31]	41	Tablet/Touchscreen	2014
Immersive Virtual Reality-Based Cognitive Intervention for the Improvement of Cognitive Function, Depression, and Perceived Stress in Older Adults With Mild Cognitive Impairment and Mild Dementia: Pilot Pre-Post Study [19]	31	HMD	2022
Evaluation of a cognition-sensitive spatial virtual reality game for Alzheimer’s disease [32]	30	Laptop	2024
Enhancing prompt perception in dementia: a comparative study of mixed reality cue modalities [23]	22	HMD	2024
Treatment of Alzheimer’s, Cognitive, Chronic Pain Rehabilitation, Depression and Anxiety disorders in One System for Elderly Using VR [33]	20	Monitor	2018
A Virtual Reality App for Physical and Cognitive Training of Older People With Mild Cognitive Impairment: Mixed Methods Feasibility Study [34]	20	HMD	2021
The Long-term Effects of Immersive Virtual Reality Reminiscence in People With Dementia: Longitudinal Observational Study [35]	20	HMD	2022
2D Virtual Reality-Based Exercise Improves Spatial Navigation in Institutionalized Non-robust Older Persons: A Preliminary Data Report of a Single-Blind, Randomized, and Controlled Study [36]	14	TV	2021
Effect of Spatial Disorientation in a Virtual Environment on Gait and Vital Features in Patients with Dementia: Pilot Single-Blind Randomized Control Trial [20]	13	Projection Screen	2020
Lessons Learned from a Human-Centered Design of an Immersive Exergame for People with Dementia [37]	11	HMD	2021
Does Practicing with a Virtual Reality Driving Simulator Improve Spatial Cognition in Older Adults? A Pilot Study [38]	11	HMD	2020
Cognitive Training Using Fully Immersive, Enriched Environment Virtual Reality for Patients With Mild Cognitive Impairment and Mild Dementia: Feasibility and Usability Study [39]	11	HMD	2020
Feasibility study of the BrightBrainer™ integrative cognitive		Laptop	
rehabilitation system for elderly with dementia [40]	10	TV	2015
Virtual Reality for Persons with Dementia: an Exergaming Experience [41]	10	Projection Screen	2012
Effects of a virtual group cycling experience on people living with dementia: A mixed method pilot study [42]	10	TV	2020
A pilot study and brief overview of rehabilitation via virtual environment in patients suffering from dementia [43]	10	HMD	2018
Enabling Patients with Neurological Diseases to perform Motor-Cognitive Exergames under Clinical Supervision for Everyday Usage [44]	8	HMD	2020
Immersive Virtual Reality Exergames for Persons Living With Dementia: User-Centered Design Study as a Multistakeholder Team During the COVID-19 Pandemic [45]	7	HMD	2022
Using exergames to train patients with dementia to accomplish daily routines [46]	7	HMD	2020
Game Design for Users with Constraint: Exergame for Older Adults with Cognitive Impairment [47]	6	HMD	2018
Virtual Reality Exergames for People Living with Dementia Based on Exercise Therapy Best Practices [48]	6	HMD	2018
Participatory design and evaluation of virtual reality games to promote engagement in physical activity for people living with dementia [49]	6	HMD	2020
Development of Serious Game Theory Framework in Virtual Reality for Alzheimer’s Patients [50]	2	TV	2024
Two-week virtual reality training for dementia: Single case feasibility study. [51]	1	HMD	2014
An Online Cognitive Intervention Tool for the Patients with Mild Cognitive Impairment using Virtual Reality [52]	not reported	HMD	2021
Memory Journalist: Creating Virtual Reality Exergames for the Treatment of Older Adults with Dementia [53]	not reported	not reported	2020

### Appendix A.1. Search Query for Scopus Database

**Listing A1.** Search query for Scopus Database.
TITLE-ABS-KEY((“dementia” OR “Alzheimer’s disease”) AND (“video games” OR “computer games” OR “exergames” OR “serious games”) AND (“augmented reality” OR “virtual reality”))

### Appendix A.2. Applied Inclusion and Exclusion Criteria

**Table A3:** Applied inclusion and exclusion criteria.

Criterion Type	Description
**Exclusion Criteria**	A valid DOI was not provided, and one could not be identified despite further investigation. Review articles were excluded to ensure a consistent methodology, enhancing the comparability of the findings. The publication was not available in English. The full text for the publication was unavailable on Scopus, PubMed, and APA Psyc (accessed via Ovid Full Text). The full text for the publication was not in the public domain. The presented study only focused on assessing dementia rather than addressing its treatment. The publication was a conference poster or abstract.
**Inclusion Criteria**	Medical: The participants had at least one of the following neurological impairments: MCI, dementia, or Alzheimer’s. Technical: The intervention integrated video gaming, exergaming, and gamification using augmented reality (AR) and virtual reality (VR). Data: The publication provided the necessary data to investigate the defined variables. The publication addressed treatments or the improvement of participants’ living conditions, such as their quality of life or mood, rather than merely assessing and screening for neurological conditions. The full text was accessible or could be obtained.

### Appendix A.3. Definition of Immersion Level

The three axes are defined by Milgram as follows.

- The Extent of World Knowledge (EWK) (the real world (AR) vs. the virtually modeled world (VR)).
- The Reproduction Fidelity (RF) (the modeling quality: wireframes vs. 3D, real-time modeling).
- The Extent of Presence Metaphor (EP) (how real you feel in the world: 2D vs. 3D VR).

The Extent of World Knowledge (EWK) is used to classify a system as AR (with limited World Knowledge) or VR (with perfect World Knowledge). The EWK axis is not essential to the results of this study and was therefore removed from the equation. We adapted the other two axes to design a system classifying the user’s level of immersion. We split the Extent of Presence into two variables and classified them from low to high (1–3). The first subcategory, EP: Delivery, covered what kind of hardware was used in the study and in what way the content was delivered to the user. The second subcategory was EP: Controls, which concerned the user’s interaction with the virtual content regarding the controllers, sensors, and input mechanisms used. The two subcategories were combined to form the Extent of Presence Axis. The second axis, the Reproduction Fidelity, represented the quality of the content delivered to the user. It included ratings for the design and composition, as well as the resolution and overall quality of the content. This axis was measured on a scale from simple to realistic (1–6).

- The rating of the *Extent of Presence: Delivery* variable from 1 to 3 (low, medium, high).
- The rating of the *Extent of Presence: Controls* variable from 1 to 3 (low, medium, high).
- The rating of the *Reproduction Fidelity* from 1 to 6 (from simple to realistic).
